# Long-term results from a randomized phase II trial of neoadjuvant combined-modality therapy for locally advanced rectal cancer

**DOI:** 10.1186/1748-717X-5-88

**Published:** 2010-09-29

**Authors:** Vaneja Velenik, Irena Oblak, Franc Anderluh

**Affiliations:** 1 Department of Radiotherapy, Institute of Oncology, Ljubljana, Slovenia

## Abstract

**Background:**

This study evaluated the effectiveness and safety of preoperative chemoradiotherapy with capecitabine in patients with locally advanced resectable rectal cancer. This report summarizes the results of the phase II study together with long-term (5-year) follow-up.

**Methods:**

Between June 2004 and January 2005, 57 patients with operable, clinical stage II-III adenocarcinoma of the rectum entered the study. Radiation dose was 45 Gy delivered as 25 fractions of 1.8 Gy. Concurrent chemotherapy with oral capecitabine 825 mg/m^2 ^twice daily was administered during radiotherapy and at weekends. Surgery was scheduled 6 weeks after the completion of the chemoradiotherapy. Patients received four cycles of postoperative chemotherapy comprising either capecitabine 1250 mg/m^2 ^bid days 1-14 every 3 weeks or bolus i.v. 5-fluorouracil 425 mg/m^2^/day and leucovorin 20 mg/m^2^/day days 1-5 every 4 weeks (choice was at the oncologist's discretion). Study endpoints included complete pathological remission, proportion of R0 resections and sphincter-sparing procedures, toxicity, survival parameters and long-term (5-year) rectal and urogenital morbidity assessment.

**Results:**

One patient died after receiving 27 Gy because of a pulmonary embolism. Fifty-six patients completed radiochemotherapy and had surgery. Median follow-up time was 62 months. No patients were lost to follow-up. R0 resection was achieved in 55 patients. A complete pathological response was observed in 5 patients (9.1%); T-, N- and overall downstaging rates were 40%, 52.9% and 49.1%, respectively. The 5-year overall survival rate, recurrence-free survival, and local control was 61.4% (95% CI: 48.9-73.9%), 52.4% (95% CI: 39.3-65.5%), and 87.4% (95% CI: 75.0-99.8%), respectively. In 5 patients local relapse has occurred; dissemination was observed in 19 patients and secondary malignancies have occurred in 2 patients. The most frequent side-effect of the preoperative combined therapy was dermatitis (grade 3 in 19 patients). The proportion of patients with severe late (SOMA grade 3 and 4) rectal, bladder and sexual toxicity was 40%, 19.2% and 51.7%, respectively.

**Conclusions:**

This study confirms data from other non-randomised studies that capecitabine-based preoperative chemoradiation is a feasible treatment option for locally advanced rectal cancer, with positive 5-year overall survival, recurrence-free survival, and local control rates.

## Introduction

Surgical resection remains the cornerstone of treatment for patients with stage II or III rectal cancer. However, curative resection is not always possible, and local relapses or metastases occur even after high-quality surgery. The use of a multidisciplinary approach, which integrates surgery, radiotherapy and chemotherapy, has become of increasing importance in this type of cancer.

For a number of years now preoperative (neoadjuvant), rather than postoperative, radiotherapy has been shown to be effective at reducing local relapses in a variety of cancer types [1-6]. In locally advanced rectal cancer, the addition of 5-fluorouracil (5-FU) to preoperative radiotherapy has been shown to improve pathological complete response rate, tumour downstaging [7] and locoregional control [8,9] compared with radiotherapy alone. Furthermore, preoperative chemoradiotherapy improves locoregional control with less toxicity when compared with postoperative radiochemotherapy [10]. Thus, preoperative radiochemotherapy with continuous infusional 5-FU has become the standard of care in rectal cancer, especially in tumours of the lower and middle rectum.

The oral fluoropyrimidine capecitabine has demonstrated efficacy comparable with intravenous 5-FU in metastatic colorectal cancer as well as in the adjuvant setting in colon cancers [11-15]. Capecitabine has also been investigated in a variety of protocols in rectal and other gastrointestinal cancers in combination with radiotherapy [16], with equivalence of capecitabine plus radiotherapy and 5-FU plus radiotherapy as preoperative therapy in locally advanced rectal cancer being demonstrated in the systematic review by Saif et al. [17].

A recent retrospective analysis from a single centre compared preoperative capecitabine to infusional 5-FU, combined with radiotherapy: once again, capecitabine showed more favourable results and higher downstaging rates [18].

The aim of this study was to evaluate the effectiveness and safety of preoperative chemoradiotherapy with capecitabine in patients with locally advanced rectal cancer. Here we summarize the results of the phase II study together and provide long-term (5-year) follow-up data.

## Patients and Methods

### Design and inclusion criteria

The trial design, eligibility criteria, treatment and initial outcome variables have been published previously in detail [19]. In brief, the trial included patients with histologically confirmed locally advanced non-metastatic resectable rectal cancer. Inclusion criteria were clinical stage II or III [UICC TNM classification]; no prior radiotherapy and/or chemotherapy; World Health Organization (WHO) performance status <2; age at diagnosis of ≥18 years; and adequate bone marrow, liver, renal and cardiac function (no history of ischemic heart disease). A history of prior malignancy other than non-melanoma skin cancer or in situ carcinoma of the cervix rendered the patient ineligible.

Prior to treatment, all patients received detailed oral and written information on the treatment protocol and possible side effects, and signed an informed consent. The trial was approved by the ethic committees of the Institute of Oncology, Ljubljana, Slovenia and of the Republic of Slovenia and was in agreement with the Declaration of Helsinki.

### Treatment protocol

Radiotherapy was delivered using 15 MV photon beams and four-field box technique, once per day, 5 days a week for 5 weeks. The small pelvis received 45 Gy in 25 fractions over 5 weeks. Three-dimensional CT-based treatment planning was performed. Patients were treated in the prone position with a full bladder during irradiation, and no devices were used to displace the small bowel out of the irradiated volume. A multileaf collimator was used for shaping the fields and for the protection of normal tissues.

Chemotherapy with capecitabine was administered concomitantly with radiotherapy at a dose of 825 mg/m^2 ^twice daily (bid) during the whole period of radiotherapy (days 1-33) without weekend breaks. Capecitabine doses were given 12 hours apart with one of the doses being taken 2 hours prior to irradiation. If radiotherapy was interrupted chemotherapy was not administered.

Definitive surgery was scheduled 6 weeks after the completion of the chemoradiotherapy. Surgical management included a sphincter preservation approach whenever possible, using the total mesorectal excision technique.

Four courses of chemotherapy were planned postoperatively. This comprised either capecitabine 1250 mg/m^2 ^bid on days 1 to 14 every 3 weeks for 4 cycles or bolus i.v. 5-fluorouracil 425 mg/m^2^/day and leucovorin 20 mg/m^2^/day on days 1 to 5 of each cycle repeated every 4 weeks. The choice of post-operative chemotherapy was left to the oncologist's discretion.

During preoperative treatment, patients were evaluated weekly for acute toxicity and compliance with the protocol. Clinical examination and complete blood count were performed and body weight was measured. Toxic side effects were assessed according to National Cancer Institute Common Toxicity Criteria (NCI-CTC) (version 2.0) [20]. Patients were followed every three month for the first two years after the last cycle of adjuvant chemotherapy and thereafter every six month up to 5th year.

The primary endpoint of the study was pathological complete response (pCR) rate. Secondary endpoints included the proportion of R0 resections, sphincter-sparing procedures, toxicity evaluation, survival parameters and long-term rectal and urogenital morbidity assessment. To assess long-term rectal and urogenital morbidity, all patients still alive, without recurrence of the disease and with a minimum follow up of 1 year were asked to complete a questionnaire about their rectal, voiding and sexual function, which was assessed using the Subjective, Objective, Management and Analytic/Late Effects on Normal Tissues scale (SOMA/LENT) [21].

### Statistics

The study aimed to evaluate whether a 12% pCR rate could be produced using this treatment approach. Setting 4% as the lowest pCR rate of interest, and with an alpha error of 5% and a power of 80%, at least 55 evaluable patients were needed.

Overall survival was defined as the time from inclusion to the date of death from any cause or to the date of last follow-up. Relapse-free survival was defined as the time from inclusion to the first occurrence of disease relapse (local or distant), death or date of last follow-up.

The Kaplan-Meier method was used to estimate the rates of overall survival, relapse-free survival and local relapse-free survival. A subgroup analysis was performed regarding relapse-free survival and the parameters that were investigated included sex, age, tumour location in the rectum (low, middle, upper third), type of surgical procedure (abdominoperineal amputation, sphincter sparing), and pathological T and N status. The log-rank test was used to test the significance between the subgroups for this endpoint. The cumulative incidence approach was used to estimate the rates for disease specific mortality, local recurrence and distant metastasis.

Statistical analysis was performed using the SPSS statistical software package, version 12 (SPSS Inc., Chicago, IL, USA).

## Results

### Patients' baseline characteristics

**Figure 1:**
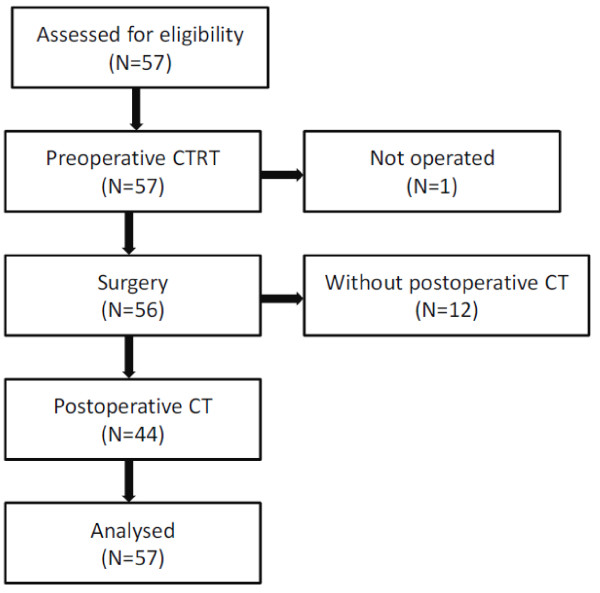
**Distribution of patients through the trial**.

Between June 2004 and January 2005, 57 patients entered the study. The study population has been described elsewhere [19]. Briefly, median age was 67 years, 75.5% were males, and 63.2% presented with stage III disease. The WHO performance status was 1 in 12.3% of patients. The median distance of the tumour from the anal verge was 5.5 cm (range 1-12 cm), and in 49.1% of patients the primary tumour was sited ≤5 cm from the anal verge. The flow of the patients through the trial is shown in Figure 1.

### Neoadjuvant therapy

One patient died after receiving 27 Gy of radiotherapy as a result of a pulmonary embolism. The remaining 56 patients (98%) completed the preoperative chemoradiotherapy according to the treatment protocol. The median preoperative treatment duration was 33 days. Preoperative chemoradiotherapy grade 3 toxicity comprised radiodermatitis (19/56 patients; 33.9%), diarrhea (2/56 patients; 3.6%), proctitis (1/56 patients; 1.8%), infection (1/56 patients; 1.8%), impaired heart function (1/56 patients; 1.8%), and leucopenia (1/56 patients; 1.8%).

### Surgery

Surgery was performed following chemoradiotherapy after a median of 45 days. In one patient only explorative laparotomy was performed as the tumour was deemed to be inoperable. As determined by histopathological examination of surgical specimens, the resection was radical (R0) in 54 patients. In one patient, microscopic foci of cancer cells were found in the radial surgical margin (R1 resection). The overall sphincter preservation rate was 65.5% and in the 27 patients where the tumour was located ≤5 cm of the anal verge the rate was 37%.

Post surgery, one patient died because of sepsis during the early perioperative period. The most frequent perioperative complication was delayed wound healing (12/56 patients; 21.8%). Re-hospitalisation was necessary for 7 patients; 2 underwent another operation because of anastomotic leak and ileus, respectively. Late surgical morbidity included urosepsis (n = 1; 1.8%), pararectal abscess (n = 1; 1.8%), and in 3 patients reoperation was required (intra-abdominal abscess, enterocutane fistula and stoma occlusion).

Thirty-six of the 39 patients (92.3%) returned the SOMA/LENT questionnaire. The proportion of patients with severe late (SOMA grade 3 and 4) rectal, bladder and sexual toxicity was 40%, 19.2% and 51.7%, respectively. More details on late toxicity have been reported previously [22].

### Tumour response

Tumour response was evaluated in 55 patients who had definitive surgery. A complete pathological response was observed in 5 patients (9.1%); T-, N- and overall downstaging rates were 40%, 52.9% and 49.1%, respectively.

### Adjuvant chemotherapy

Eleven patients never received adjuvant chemotherapy either because of: death during the perioperative period for 1 patient (1.8%); cardiotoxicity experienced in preoperative treatment in 1 patient (1.8%); postoperative pathological echocardiogram in 1 patient (1.8%); surgical complications in 5 patients (9%); and time from operation more than 8 weeks in 3 patients (5.5%). Postoperative chemotherapy was administered to 44 of 55 radically operated patients (80%); 18/44 (40.9%) patients received bolus 5-fluorouracil/leucovorin (5-fluorouracil 425 mg/m^2 ^plus leucovorin 20 mg/m^2 ^for 5 days, every 28 days) - 17 patients received 4 cycles and 1 patient received 3 cycles because of a grade 3urinary infection; 26/44 patients (59.1%) received capecitabine 1250 mg/m² for 14 days, every 21 days - 16 patients received 4 cycles, 1 patient received 1 cycle because of grade 3cardiotoxicity and 1 patient received 3 cycles because of prolonged grade 2 leukopenia. Eleven (20%) radically operated patients did not received postoperative chemotherapy. The choice of chemotherapy regimen was at the discretion of the investigator.

### Treatment outcome

**Figure 2:**
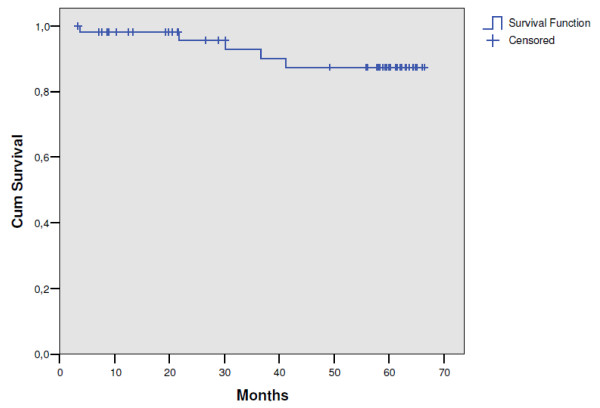
**Local recurrence-free survival (n = 56)**.

**Figure 3:**
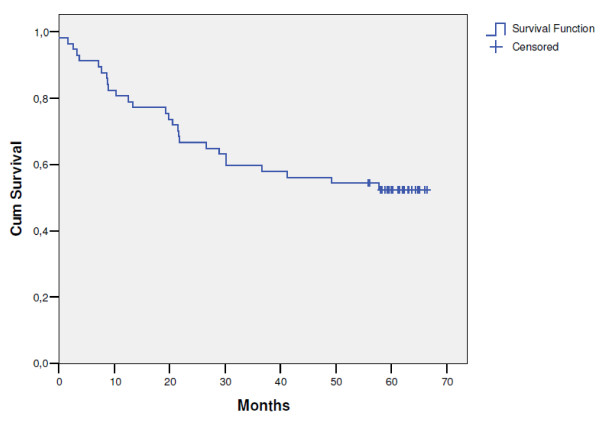
**Recurrence-free survival (n = 57)**.

**Figure 4:**
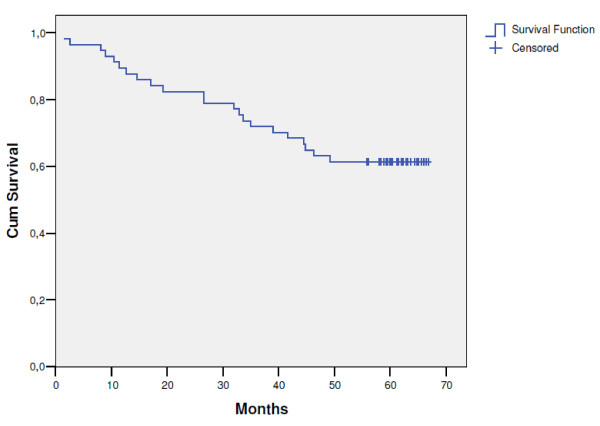
**Overall survival (n = 57)**.

The median follow-up time for patients still alive in this trial was 62 months. The median time to disease recurrence was 19.5 months (range: 3.3-58 months). The pattern of first recurrence was predominantly distant metastases, which were observed in 18 patients (33.3%). Local progression was the site of failure in 4 patients (7.4%), whereas 1 patient (1.8%) had synchronous local and distant disease. The 5-year local control rate was 87.4% (95% CI: 75.0-99.8) (Figure 2). In 5 patients local relapse occurred; dissemination was observed in 19 patients and secondary malignancies occurred in 2 patients. Nineteen patients (35.2%) developed distant metastases of which two were discovered during surgery. The latest local and distant failures were observed after 42 and 58 months, respectively. Relapse-free survival rate was 52.4% (95% CI: 39.3-65.5) (Figure 3). It was found that survival was independent of gender (p = 0.47), age (p = 0.58), tumour location in the rectum (p = 0.32), type of operation (p = 0.22) and pathological T status (p = 0.35), but it was significantly better in patients with pathological negative nodes than in patients with positive nodes (66.5% vs. 36.4%; p = 0.01). As of April 2010, 22 patients (38.6%) of the entire study population have died. One patient (1.8%) died of treatment complications, 15 (26.3%) died of rectal cancer, 1 (1.8%) of a second primary cancer and the remaining 5 patients of other causes (8.7%). The 5-year overall survival rate was 61.4% (95% CI: 48.9-73.9) (Figure 4).

## Discussion

Patients with locally advanced stage II/III rectal cancer should preferably receive some form of neoadjuvant treatment to downstage the tumour and enable a potentially curative resection. Fluoropyrimidine-based chemoradiation is currently a well-accepted approach in the management of locally advanced rectal cancer with several retrospective and prospective trials suggesting that preoperative capecitabine is at least equivalent to infusional 5-FU when combined with radiotherapy, and may improve tumour downstaging. Since 2009, capecitabine has been recommended by the US National Comprehensive Cancer Network as an acceptable alternative to 5-FU in this setting [20].

In the initial part of this study the complete pathological response rate was 9.1%, tumour (T), lymph nodes (N), and overall downstaging rates were 40%, 52.9%, and 49.1%, respectively, the total sphincter preservation rate was 65.5% (36 out of 55 patients) and the rate in 27 patients with tumours located within 5 cm of the anal opening was 37% (10 out of 27 patients) [19]. These findings are similar to some other studies using single-agent capecitabine, such as Dunst et al. [23] and Craven et al [24] where the complete pathological response rates were 7% and 9%, respectively; that said, in small studies with oral capecitabine the complete pathological response rate has ranged from 0 to 31% [23-39], while in the study by Kim et al. [40], which is one of the largest studies to date, the rate was 12%. It is noteworthy that the findings from this study are comparable to studies using single-agent 5-FU [41].

Overall, a number of phase II studies with comparable designs to that used here have shown favorable toxicity profiles and pathological complete response rates with capecitabine chemoradiotherapy [25-39]. Thus, these studies have shown comparable results suggesting that the combination of capecitabine and pelvic radiation is safe and effective and that capecitabine can replace continuous infusional 5-FU. The toxicity of the combination of capecitabine chemotherapy and radiotherapy was low, as expected, and has been reported elsewhere [22]. The most frequent side-effect of the preoperative combined therapy was dermatitis (grade 3 in 19 patients). These safety data compare favorably with the results of other phase I/II studies in rectal cancers. Long-term toxicity found the proportion of patients with severe late (SOMA grade 3 and 4) rectal, bladder and sexual toxicity was 40%, 19.2% and 51.7%, respectively.

In 2008, Dunst et al. [23] was the first to report long-term follow-up on survival and local control in patients with locally advanced rectal cancer having undergone neoadjuvant capecitabine-based chemoradiotherapy followed by surgery. Here, in what we believe to be only the second long-term follow-up, in 55 patients with locally advanced rectal cancer who underwent surgery considered curative the 5-year overall survival rate, recurrence-free survival rate, and local control rate were 61.4%, 52.4%, and 87.4%, respectively. The 5-year overall survival reported here is similar to the 65% reported by Dunst et al. [23]. However, the rate of local recurrence reported here (12.3%) was lower than the cumulative risk of local recurrence after 5 years reported by Dunst et al. [21] (17%).

While many questions regarding the use of adjuvant therapy in patients with locally advanced rectal cancer have yet to be answered, and data regarding capecitabine in this setting are limited, it is clear that capecitabine is an effective and more convenient alternative to 5-FU when combined with radiotherapy in the preoperative treatment of patients with locally advanced rectal cancer. A number of on-going trials are taking place that incorporate capecitabine as an integral part of the design with the aim to refine the management of this patient group and, as such, is likely to assume a major role in the treatment of rectal cancer in the future.

## Conclusion

The results of this long-term study confirm data from other non-randomised studies that capecitabine-based preoperative chemoradiation is a feasible treatment option for locally advanced rectal cancer, with positive 5-year overall survival, recurrence-free survival, and local control rates. Complete pathological response rates were similar to those reported with single-agent 5-FU.

## Abbreviations

5-FU: 5-fluorouracil.

## Competing interests

The authors declare that they have no competing interests.

## Authors' contributions

VV: contributions to conception and design, acquisition of data, analysis and interpretation of data; involvement in drafting and reviewing the manuscript. IO: contributions to acquisition of data. FA: contributions to acquisition of data, analysis and interpretation of data; involvement in drafting and reviewing the manuscript. All authors have read and approved the final version of the manuscript.
